# Effects of Subinhibitory Concentrations of Ceftaroline on Methicillin-Resistant *Staphylococcus aureus* (MRSA) Biofilms

**DOI:** 10.1371/journal.pone.0147569

**Published:** 2016-01-22

**Authors:** María Lázaro-Díez, Sara Remuzgo-Martínez, Cristina Rodríguez-Mirones, Felix Acosta, Jose M. Icardo, Luis Martínez-Martínez, José Ramos-Vivas

**Affiliations:** 1 Servicio de Microbiología, Hospital Universitario Marqués de Valdecilla and Instituto de Investigación Marqués de Valdecilla (IDIVAL), Santander, Cantabria, Spain; 2 Red Española de Investigación en Patología Infecciosa (REIPI), Instituto de Salud Carlos III, Madrid, Spain; 3 Grupo de Investigación en Acuicultura, Universidad de Las Palmas de Gran Canaria, Arucas, Gran Canaria, Spain; 4 Departamento de Anatomía y Biología Celular, Universidad de Cantabria, Santander, Cantabria, Spain; 5 Departamento de Biología Molecular, Universidad de Cantabria, Santander, Cantabria, Spain; Institut National de la Recherche Agronomique, FRANCE

## Abstract

Ceftaroline (CPT) is a novel cephalosporin with *in vitro* activity against *Staphylococcus aureus*. Ceftaroline exhibits a level of binding affinity for PBPs in *S*. *aureus* including PBP2a of methicillin-resistant *S*. *aureus* (MRSA). The aims of this study were to investigate the morphological, physiological and molecular responses of MRSA clinical strains and MRSA biofilms to sub-MICs (1/4 and 1/16 MIC) of ceftaroline by using transmission, scanning and confocal microscopy. We have also used quantitative Real-Time PCR to study the effect of sub-MICs of ceftaroline on the expression of the staphylococcal *icaA*, *agrA*, *sarA* and *sasF* genes in MRSA biofilms. In one set of experiments, ceftaroline was able to inhibit biofilm formation in all strains tested at MIC, however, a strain dependent behavior in presence of sub-MICs of ceftaroline was shown. In a second set of experiments, destruction of preformed biofilms by addition of ceftaroline was evaluated. Ceftaroline was able to inhibit biofilm formation at MIC in all strains tested but not at the sub-MICs. Destruction of preformed biofilms was strain dependent because the biofilm formed by a matrix-producing strain was resistant to a challenge with ceftaroline at MIC, whereas in other strains the biofilm was sensitive. At sub-MICs, the impact of ceftaroline on expression of virulence genes was strain-dependent at 1/4 MIC and no correlation between ceftaroline-enhanced biofilm formation and gene regulation was established at 1/16 MIC. Our findings suggest that sub-MICs of ceftaroline enhance bacterial attachment and biofilm formation by some, but not all, MRSA strains and, therefore, stress the importance of maintaining effective bactericidal concentrations of ceftaroline to fight biofilm-MRSA related infections.

## Introduction

Infections caused by methicillin-resistant strains of *S*. *aureus* (MRSA) range from those of the skin and surgical sites, infections relating to catheters and prosthetic implants, to bacteremia, endocarditis and pneumonia [1]. The ability of *S*. *aureus* to form biofilms is an important characteristic which complicates infections due to MRSA, especially those associated with foreign materials such as catheters and implants [2]. Biofilms can be defined as a structured community of bacterial cells enclosed in a self-produced polymeric matrix and adherent to an inert or living surface. Growth in biofilm enables bacterial populations to survive better in hospital environments and during host infections (i.e. in the presence of antibiotics), increasing the probability of causing nosocomial infections [3–5]. Among *S*. *aureus* strains, production of a polysaccharide adhesin, termed polysaccharide intercellular adhesin (PIA) or polymeric *N*-acetyl-glucosamine (PNAG), by *ica* operon-encoded enzymes is currently the best-understood mechanism of biofilm development, that may contribute to chronic infections [6, 7]. However, MRSA biofilm formation in strains that do not express *ica* genes have also been described. In this case, another proteins such as biofilm-associated proteins (Bap) or fibronectin-binding proteins (FnBPs) are responsible for cell aggregation and, therefore, of biofilm persistence and maturation [8]. In addition to chronic infections, *S*. *aureus* can cause acute diseases, many of which are mediated by the ability of this pathogen to produce surface structures that facilitate tissue colonization, and/or extracellular toxins. Production of these factors is regulated by a quorum-sensing mechanism, predominantly under the control of the accessory gene regulator (*agr*) operon where the transcription factor AgrA controls the expression of all the virulence factors under the control of the *agr* system [9, 10]. Furthermore, the *sasF* gene, which encodes a putative surface anchored protein (SasF) with significant homology to the biofilm-associated protein SasG and the Staphylococcal accessory regulator (*sarA*), one of the central elements related to the regulation of virulence factors, could play important roles in MRSA biofilm formation [11, 12].

The effects of subinhibitory levels of antibiotics on bacteria have long been recognized, especially with respect to their action on target cell morphology [13, 14]. Also, different studies have shown that some antibiotics, when present at concentrations below the minimal inhibitory concentration (MIC) can significantly induce biofilm formation in a variety of bacterial species *in vitro*, including *S*. *aureus* [15–19]. Moreover, subinhibitory concentrations (sub-MICs) of antibiotics have been examined for their ability to cause global changes in gene transcription [20]. Therefore, the effects of sub-MICs of antibiotics on microorganisms is of continuing interest to microbiologists in the clinical laboratory.

Ceftaroline (CPT), the active metabolite of the prodrug CPT-fosamil, is a novel cephalosporin with *in vitro* activity against *S*. *aureus*, including MRSA [19, 21–23]. This drug was approved in the United States for the treatment of adults with acute bacterial skin and skin structure infections and community-acquired bacterial pneumonia and by the European Medicines Agency for the treatment of patients with complicated skin and soft tissue infections and community-acquired pneumonia. Ceftaroline, unlike other compounds of the same family, exhibits a high affinity for PBPs in *S*. *aureus* including PBP2a, which is the base of their action mechanism [24], and no high-level resistance has yet been reported, despite the study of thousands of clinical isolates [25].

To the best of our knowledge, there is no report on the effects of sub-MICs of ceftaroline on MRSA biofilm formation. Since there are a number of *in vivo* circumstances where concentrations of ceftaroline may be at subinhibitory levels, the objective of this study was to evaluate the effects of sub-MICs levels of ceftaroline on MRSA biofilm formation by using transmission, scanning and confocal microscopy. Our study brought clear evidence that ceftaroline at sub-MICs significantly increases the biofilm formation capability of some MRSA clinical strains.

## Materials and Methods

### Bacterial strains

Thirteen *S*. *aureus* strains were used in this work (Table 1). All clinical isolates were obtained from blood cultures from different patients (normal service of routine) at the University Hospital Marqués de Valdecilla, Santander, Spain. All strains were resistant to oxacillin with a MIC > 16 μg/ml. Methicilin-susceptible *S*. *aureus* (MSSA) ATCC 29213 was included as an oxacillin susceptible control strain (MIC = 0.125 μg/ml) in some tests. *S*. *aureus* strains were routinely cultured on blood agar (BA) plates at 37°C. Our research did not involve human participants or samples. Despite the bacteria were obtained from blood samples, these samples were discarded later on and destroyed.

**Table 1:** Strains used in this study and MICs of ceftaroline.

Strain	^a^PFGE	^b^CLON	^c^MIC (μg/mL)
04/329	9	ST125	0.5
06/725	1	ST125	0.5
06/1156	83	ST125	0.5
06/1483	14	ST125	0.5
05/1784	7	ST5	0.25
06/2360	7	ST5	0.25
05/2369	58	ST125	0.5
04/3069	25	ST125	0.5
05/3290	1	ST125	0.5
06/3273	3	ST125	0.5
05/3291	26	ST5	0.5
06/3634	68	ST125	0.5
ATCC 29213	-	-	0.125

^a^Patterns defined for a study in our lab, considering >300 isolates.

^b^Sequence types (ST) defined by multilocus sequence typing.

^c^Each strain was tested at least in three independent experiments and modal values are shown. MIC Range: 0.015–16 μg/ml.

### MIC determination

Conventional antimicrobial susceptibility testing was performed by the standardized broth microdilution method in 96-well U-bottom plates (Sarstedt) using Mueller Hinton (MH) Broth (Difco) according to the CLSI guidelines [26].

### Transmission electron microscopy (TEM)

The bacterial cell morphology and/or morphological changes in planktonic cells induced by the MIC and sub-MICs of ceftaroline were evaluated by TEM. Planktonic cells were applied to 100 mesh Cu grids supported with carbon-coated Formvar film (Electron Microscopy Sciences) and air dried. The cells were then negatively stained with 1% phosphotungstic acid in distilled water for 20s and examined with a JEOL (JEM-1011) transmission electron microscope operating at 80 kV, and equipped with an ORIUS SC 1000 CCD camera (GATAN).

### Biofilm formation/disruption

Biofilm formation/disruption was assessed in presence of ceftaroline at MIC or sub-MICs by the crystal violet (CV) method [27], using 33% glacial acetic acid (GAA) or ethanol:acetone (80:20 v/v) as destaining solutions, in 24-well plates (Iwaki, Tokyo, Japan). MRSA strains and the MSSA strain were cultivated on BA plates for 24 hours at 37°C. Some colonies of each strain with identical morphology were suspended in physiological saline. The turbidity of the bacterial suspension was adjusted to 0.5 McFarland standard ∼10^8^ colony-forming units ml^-1^. The suspension was vortexed for at least 1 min. This inoculum was diluted 1:100 in Mueller-Hinton supplemented with 1% glucose (Sigma-Aldrich) (MH-G). On the other hand, antibiotic dilution was prepared in MH-G at different concentrations: 2 μg/ml, 1 μg/ml, 0.5 μg/ml, 0.25 μg/ml, 0.125 μg/ml, 0.06 μg/ml, and 0.03μg/ml. Finally, in each well of 24-well plates, 500 μl of bacterial inocula (final dilution 1:200) and 500 μl of the corresponding antibiotic dilution were added. Inoculated MH-G without antibiotic was included as a positive control for biofilm formation. Plates were incubated statically at 37°C for 48 hours.

In a second set of experiments, destruction of preformed biofilms (48h of growth in absence of antibiotic) by addition of different concentrations of ceftaroline (1 μg/ml, 0.5 μg/ml, 0.25 μg/ml, and 0.125 μg/ml) for 24h was evaluated.

Planktonic cells were removed and wells containing biofilms were rinsed two times with 2 ml of distilled water. The remaining adherent bacteria were stained with crystal violet (Sigma-Aldrich) (0.7% w/v solution) for 12 min. Excess stain was removed by washing twice with distilled water (with 2 ml per well). CV was extracted with 1 ml of 33% acetic acid (Panreac) and the plates were incubated at room temperature (RT) in an orbital shaker for 1 min at 400 rpm to release the dye into solution. Two samples of 100 μl were then transferred from each well to a 96-well flat-bottomed plate (Nunc™ Thermo Scientific™) and the amount of dye (proportional to the density of adherent cells) was quantified at OD_620_ using a microplate reader (Thermo Scientific Multiskan FC). For each experiment, correction for background staining was made by subtracting the value for CV bound to uninoculated control wells. Each bacterial strain was tested in duplicate, in four independent experiments for each condition (biofilm formation/disruption).

Glacial acetic acid (33%) used as destaining solution is able to release more dye from biofilms into solution, than the classical ethanol:acetone solution (80:20 v/v) (S1 Fig), so we recommend the use of this destaining solution for work with MRSA instead of the classical ethanol:acetone solution. Due to these observations, we therefore use MH-G and GAA to assess biofilm formation in our strains.

### Scanning electron microscopy (SEM)

For SEM, strains 06/1483 and 05/3291 were selected (each representing a different clone, ST125 and ST5 respectively) and grown for 48h on 24-well polystyrene plates (Nunc, Thermo Scientific) in presence of ceftaroline at MIC and sub-MICs, fixed and processed for SEM. Biofilms were processed directly inside the plates after removing culture media and washing. The entire wells were fixed with ice-cold 3% glutaraldehyde for 20 min at 4°C. Wells were then dehydrated in a graded ethanol series (30%, 50%, 70%, 90% and 100%, 10 min each), dried by the critical point method, coated with gold in a Fine coat ion sputter JFC-1100 (JEOL) and observed with an Inspect S microscope (FEI Company) working at 15 or 20 KV. Experiments were performed at least in duplicate.

### Confocal Laser Scanning Microscopy (CLSM)

Bacteria were grown in 4-well μ-chamber uncoated slides (Ibidi, Martinsried) without shaking. The assays were performed in presence of different concentrations of ceftaroline (MIC, 1/4 MIC, and 1/16 MIC) and a positive control without antibiotic. Inoculums and antibiotics were prepared as explained above. The slides were incubated at 37°C for 48h. After 48h, planktonic cells were removed by rinsing with saline (0.85% NaCl) and bacterial viability within biofilms was determined by adding 200μl of the *Ba*cLight LIVE/DEAD bacterial viability kit (Molecular Probes Inc.) per well for 25 min.

For matrix visualization, unfixed biofilms were stained with 200 μl of FilmTracer^TM^ SYPRO Ruby Biofilm Matrix (Invitrogen) per well, incubated in the dark for 30 min at RT, and rinsed with distilled water. As a positive control for biofilm matrix formation, we used a *Pseudomonas aeruginosa* clinical isolate, which produces a dense and compact biofilm with abundant extracellular matrix. For bacterial DNA staining, unfixed biofilms were stained with one drop of NucBlue (Molecular Probes) applied for 30 minutes to each well. For LIVE/DEAD a 488/561 nm excitation, 500–550/570–620 nm emission filters were used respectively. For FilmTracer^TM^ a 405 nm excitation, 662–737 nm emission filter was used. For NucBlue, a 375–390 nm excitation, 420–490 nm emission filter was used. A series of optical sections were obtained with a Nikon A1R confocal scanning laser microscope. Images were captured at random with a ×20 Plan Apo 0.75 NA objective. Reconstructions of confocal sections were assembled using the NIS-Elements 3.2 software. Z-stacks of confocal images were rendered into 3D mode using the ImageJ software.

### Gene expression from biofilms

Quantification of *gyrB*, *rRNA*, *agrA*, *icaA*, *sarA* and *sasF* mRNA levels in *S*. *aureus* biofilms exposed to sub-MICs of ceftaroline was conducted by real-time quantitative PCR. RNA was extracted from 48h MRSA biofilms using TRIzol® Max™ Bacterial RNA Isolation Kit (Invitrogen) according to the manufacturer instructions. The RNA concentration was quantified with a NanoDrop spectrophotometer and RNA purity was assessed by 260/280 and 260/230 ratios. cDNA was obtained by retrotranscription of 100 ng of the total RNA using the iScript cDNA synthesis kit (Bio-Rad) according to the manufacturer´s protocol. Real-time PCR was performed using the Sso Fast Evagreen MasterMix in a CFX96 system (Bio-Rad). Each reaction of PCR consisted in 0.4 μM of forward primer, 0.4 μM of reverse primer, 10 μL SsoFast EvaGreen supermix, nuclease free water and cDNA until 20 μL of reaction volume.

The PCR cycling program was set as follows: stage 1: 95°C for 30 sec, stage 2: 95°C for 3 sec followed by 60°C for 3 sec repeated for 40 cycles. Specificity of PCR products was determined by gel electrophoresis. Primers used for q-RT-PCR are listed in Table 2.

**Table 2:** Primer used for qRT-PCR.

Name of Genes	Forward primer (5’-3’)	Backward primer (5’-3’)	Reference
*gyrB*	TTATGGTGCTGGGCAAATACA	CACCATGTAAACCACCAGATA	[28]
*rRNA*	ATGCAAGTCGAGCGAAC	TGTCTCAGTTCCAGTGTGGC	[29]
*agrA*	CCTCGCAACTGATAATCCTTATG	ACGAATTTCACTGCCTAATTTGA	[9]
*icaA*	CTTGGATGCAGATACTATCG	GCGTTGCTTCCAAAGACCTC	[30]
*sarS*	AATACCCTCAAACTGTTAGAGC	TCACTTGAGCTAATAATTGTTCAG	[12]
*sasF*	CGTCCTCGTCACTTTGTTGA	CGAAAACAGCATCGCAAATA	[11]

### Statistics

In CV quantitative analysis, mean were calculated using eight values (two measures in four assays) ± standard error (SE). Differences in OD_620_ (biofilm formation and biofilm maintenance) were analyzed by calculating p-values based on t-test using Welch’s correction (not assume equal variances) with GraphPad Prism 3.0 statistical software. A p-value less than 0.05 was considered statistically significant.

For the real-time quantitative PCR, the threshold cycle (Ct) of each well and data acquisition were carried out by the CFX-Manager software (Bio-Rad), and the cut-off value to consider a result as positive was set to a C_t_ value of 35. The delta Ct (ΔCt) method was used for PCR single gene data analysis. The normalized (ΔCt) for each gene of interest (GOI) was calculated by substracting the mean C_t_ of the two housekeeping genes from the C_t_ of each GOI. Then the double delta C_t_ (ΔΔC_t_) for each GOI was calculated by deducting the mean ΔC_t_ of GOI in the control group (without ceftaroline) from the ΔC_t_ of each GOI. The fold-change of each GOI compared with the control group was calculated as 2^-ΔΔCt^ from the log_10_2^-ΔΔCt^. Fold-regulation represents fold-change results in a biologically meaningful way. A fold-change value greater than 1 indicates positive- or an up-regulation, and the fold-regulation is equal to the fold-change. Fold-change values less than 1 indicate negative or down-regulation, and the fold-regulation is the negative inverse of the fold-change. The p-values were analyzed by Graphpad Prism 3.0 statistical software using Student’s t-test of the C_t_ values for each gene in the ceftaroline-treated and control groups. A p-value less than 0.05 was considered statistically significant.

## Results

### MICs

Minimal inhibitory concentrations of ceftaroline are listed in Table 1. All the MRSA strains used in the study were susceptible to ceftaroline, with MICs ranging 0.25–0.5 μg/ml. MICs of ceftaroline using 24 well plates and MH-G were identical to those obtained using the classical microdilution method (96 well plates and MH as culture medium).

### Evaluation of bacterial morphology by TEM and SEM

**Fig 1:**
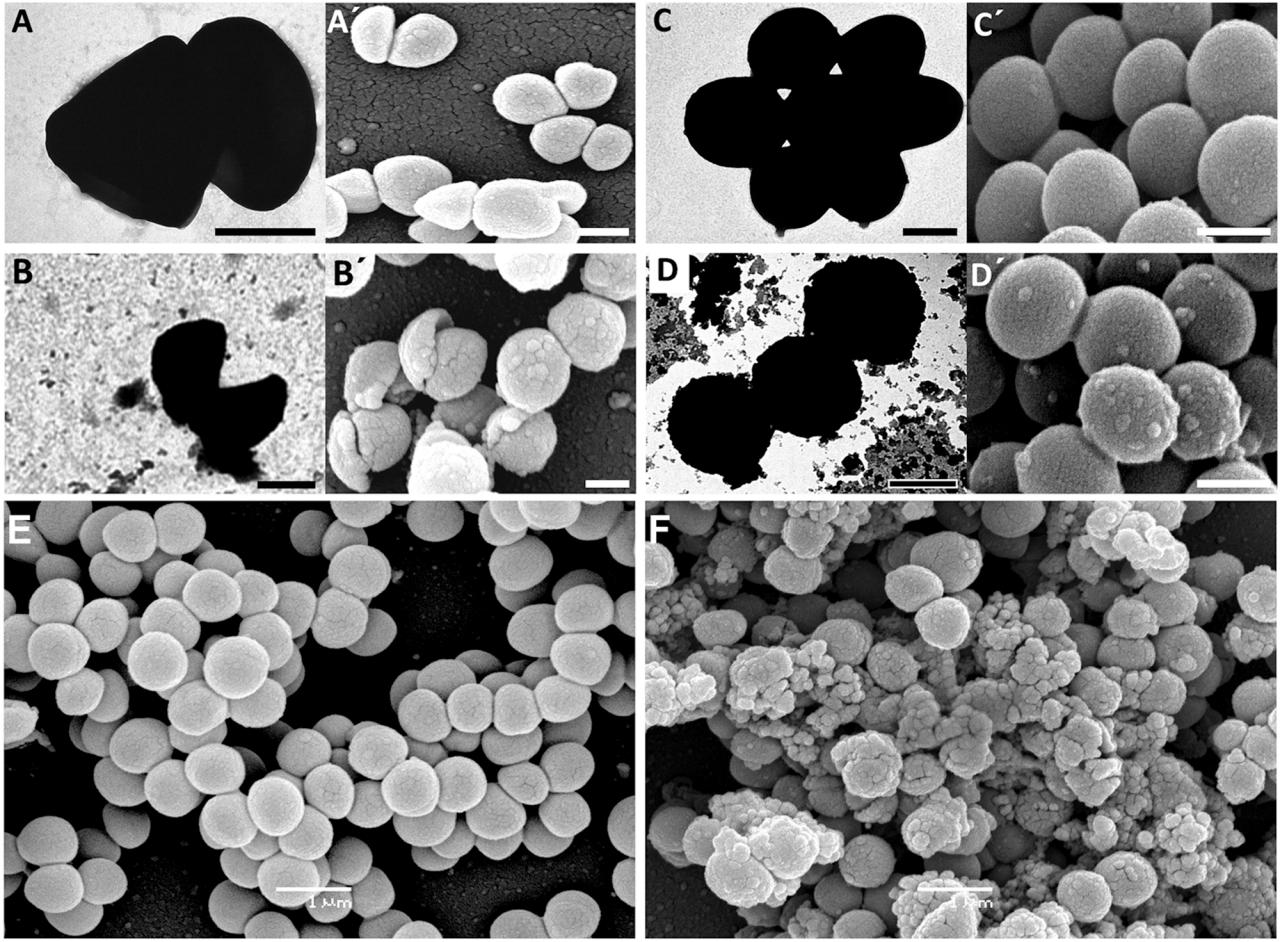
The bacterial cell morphology of MRSA treated with ceftaroline. TEM, A-D; SEM, (A´-D´). (A-A´) strain 06/1483 after exposure to 1/2 MIC of ceftaroline. (C-C´) strain 06/1483 without antibiotic. (B-B´) strain 05/3291 after exposure to 1/2 MIC of ceftaroline. (D-D´) strain 05/3291 without antibiotic. Biofilms of strains 06/1483 (E) and 05/3291 (F) exposed to 1/2 MIC of ceftaroline. Original Magnifications: (A) ×80.000; (A´) ×15.000; (B) ×50.000; (B´) ×15.000; (C) ×40.000; (C´) ×20.000; (D) ×60.000; (D´) ×20.000. (E,F) ×15.000. Scale bars, (A-D´) 1μm.

The bacterial cell morphology and/or morphological changes in planktonic cells and biofilms induced by the MIC and sub-MICs of ceftaroline were evaluated by TEM and SEM respectively. Ceftaroline causes two main types of damage in MRSA: cell-shape deformation and cell wall breakdown. Representative images for TEM and SEM in strains 06/1483 and 05/3291 are shown in Fig 1. Similar results were obtained using other strains (not shown). Structural analysis to evaluate biofilm thickness and 3D architecture was performed by SEM. High magnification of both strains shows that biofilm of strain 05/3291 grown in presence of ceftaroline forms an extracellular matrix whereas strain 06/1483 does not. Representative SEM images of biofilms at MIC and 1/2 MIC are shown in Fig 1E and 1F.

### Quantitative biofilm analysis

**Fig 2:**
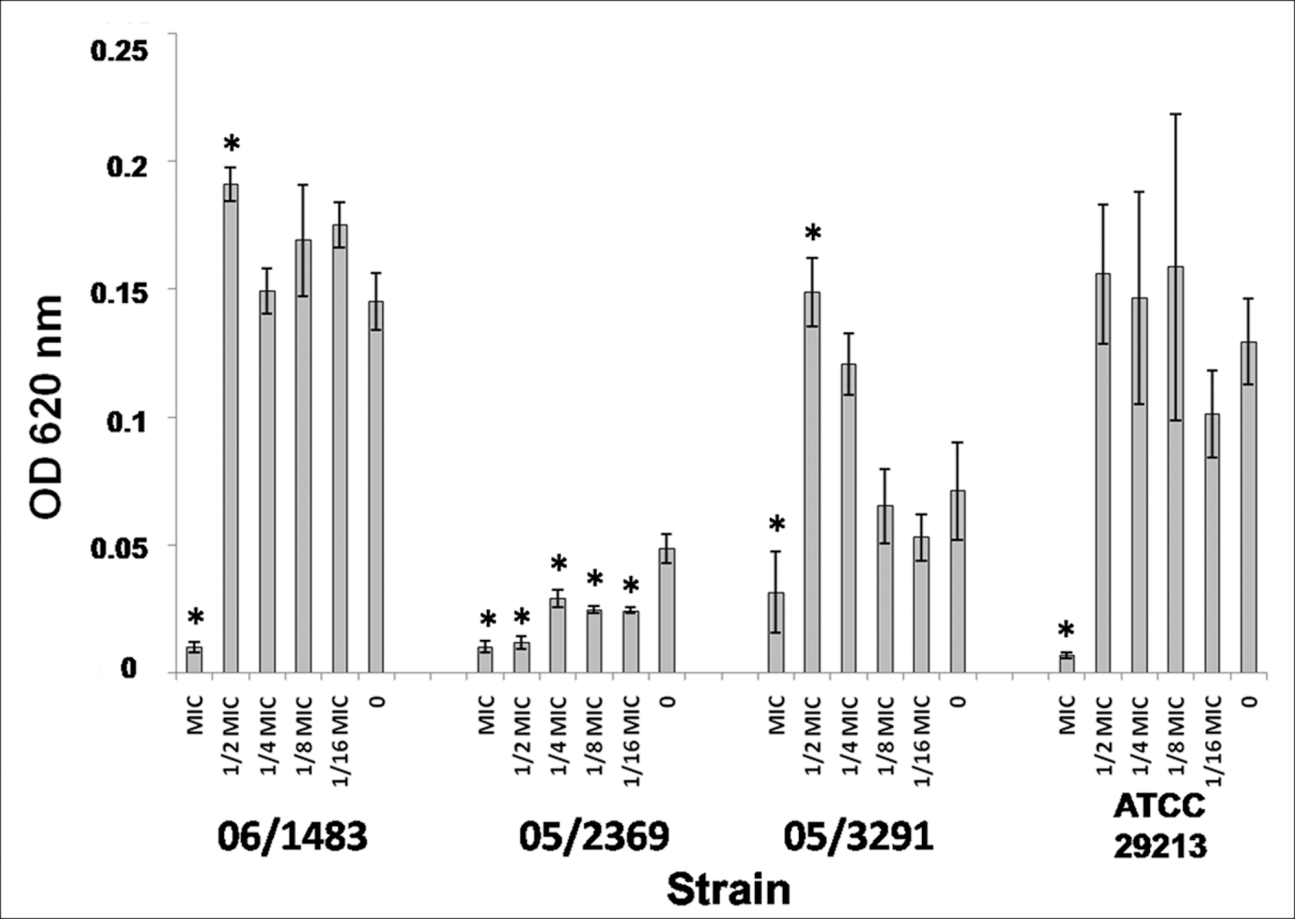
Effects of ceftaroline on biofilm formation. Biofilm formation by MRSA in presence of ceftaroline, in 24-well polystyrene plates after 48 h, assessed by crystal violet staining. Each bar indicates the mean values ± SE from four independent experiments. Strains 06/1483, 05/2369, 05/3291 were tested. ATCC 29213 was also included. 0, no antibiotic added (control). X-axis represents ceftaroline concentrations (respect to the MIC). *Indicates significant differences between the concentration tested and control.

Enhanced biofilm formation was observed when the isolates were grown in MH supplemented with 1% glucose. Three MRSA clinical isolates (strains 06/1483, 05/2369, 05/3291) and the methicillin susceptible control strain (ATCC 29213) were selected as these were good biofilm formers and representative of two different clones. For this reason, the next experiments were done using these strains. Strains were cultured in the presence of MICs and sub-MICs (1/2 MIC to 1/16 MIC) of ceftaroline for 48h, and biofilm formation was compared with strains cultured without antibiotic. Ceftaroline was able to inhibit biofilm formation at MIC in all strains tested. It is important to note a strain dependent behavior in presence of sub-MICs: whereas strain 05/2369 reduced its biofilm at the sub-MICs tested. In the other strains, the increase of biofilm formation is not statistically significant at most concentrations. Results are shown in Fig 2.

**Fig 3:**
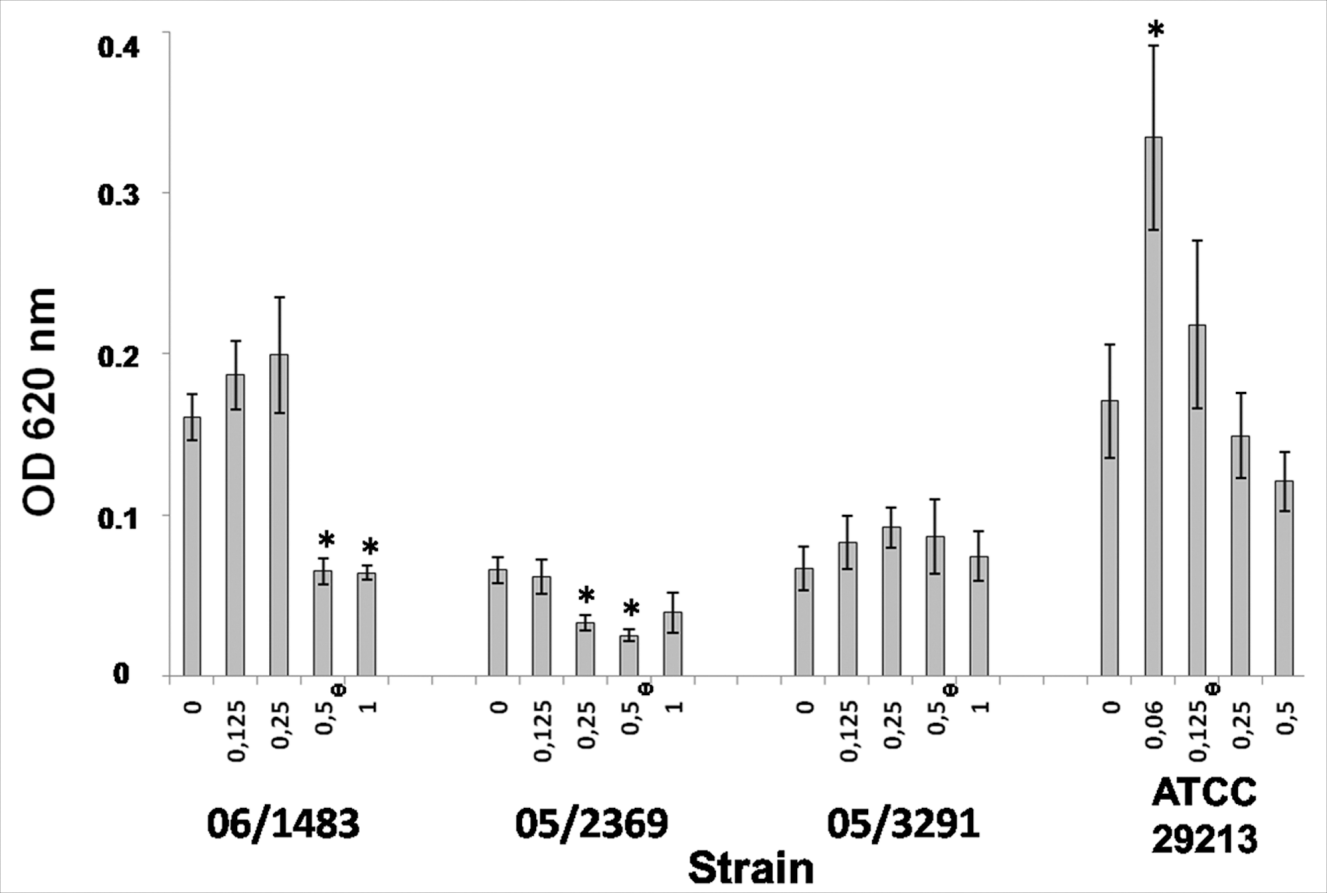
Effects of ceftaroline on biofilm disruption. Disruption of preformed biofilms by ceftaroline after growth in 24-well polystyrene plates and assessed by crystal violet staining. Each bacterial strain was tested in duplicate, and bars indicate the mean values ± SE from four independent experiments. Strains 06/1483, 05/2369, 05/3291 and ATCC 29213 were tested. 0, no antibiotic added (control). Ceftaroline concentrations (μg/ml) are indicated on the X-axis. *Indicates significant differences between the concentration tested and control. Φ Indicates the MIC for each strain.

In a second set of experiments, disruption of preformed biofilms (48h of growth in absence of antibiotic) by addition of ceftaroline at different concentrations for 24h was evaluated. Destruction of preformed biofilms was also strain dependent and ceftaroline was able to reduce, at certain concentrations, preformed biofilms in two clinical isolates: 06/1483 and 05/2369, but not in strain 05/3291. Results are shown in Fig 3.

### Structural analysis of biofilms exposed to MICs and sub-MICs by CLSM

**Fig 4:**
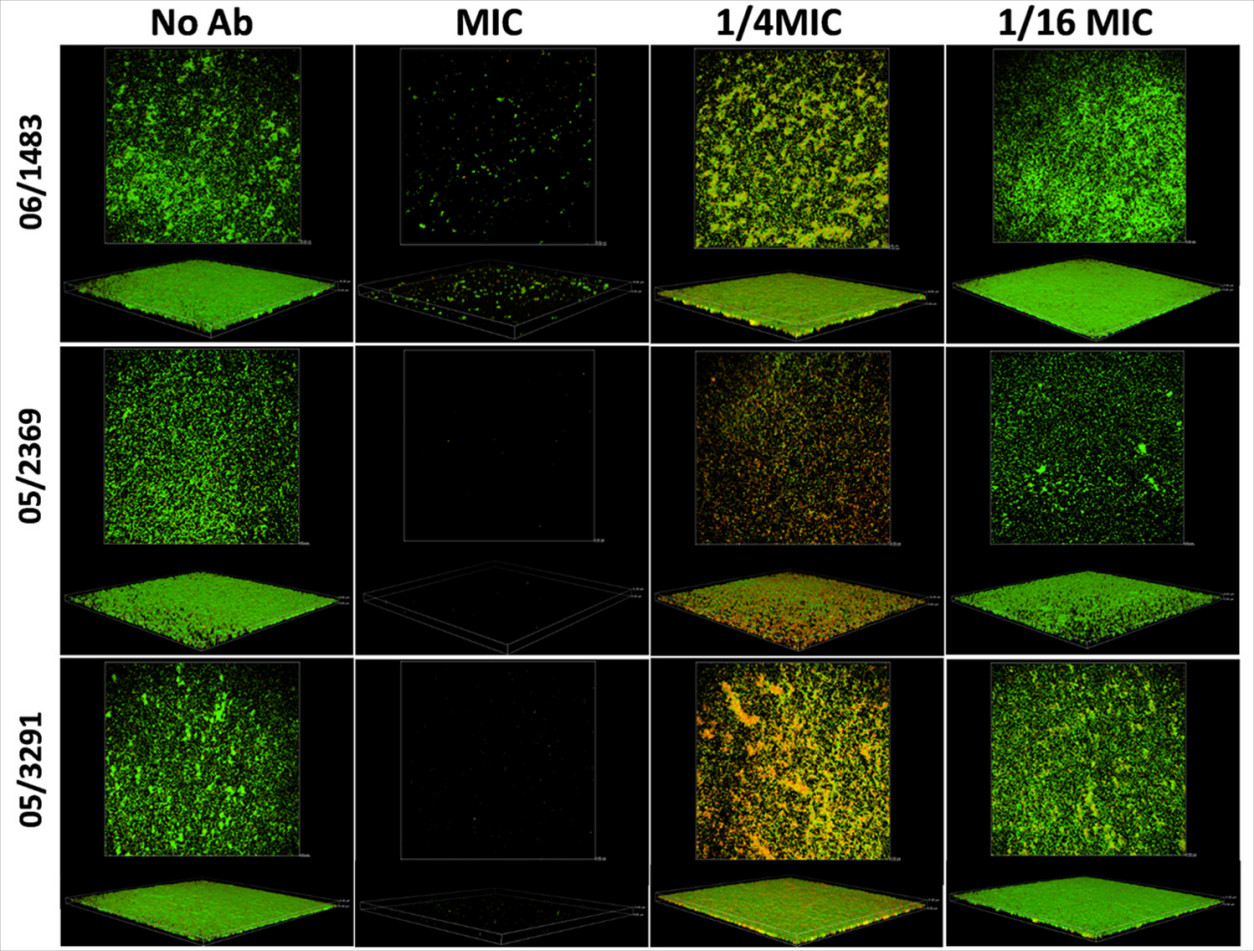
CLSM of biofilms formed by MRSA strains in presence of ceftaroline. Confocal Laser Scanning Microscopic images of strains 06/1483, 05/2369, and 05/3291 after growth on uncoated 4-well chamber slides and stained with the LIVE/DEAD viability kit. Live cells are stained green with Syto 9 dye and dead cells are stained red with propidium iodide. MIC and sub-MICs are indicated. Original magnification ×200.

Similar to SEM results were obtained after staining of MRSA biofilms exposed to ceftaroline and processed by CLSM. Representative CLSM images are shown in Fig 4.

**Fig 5:**
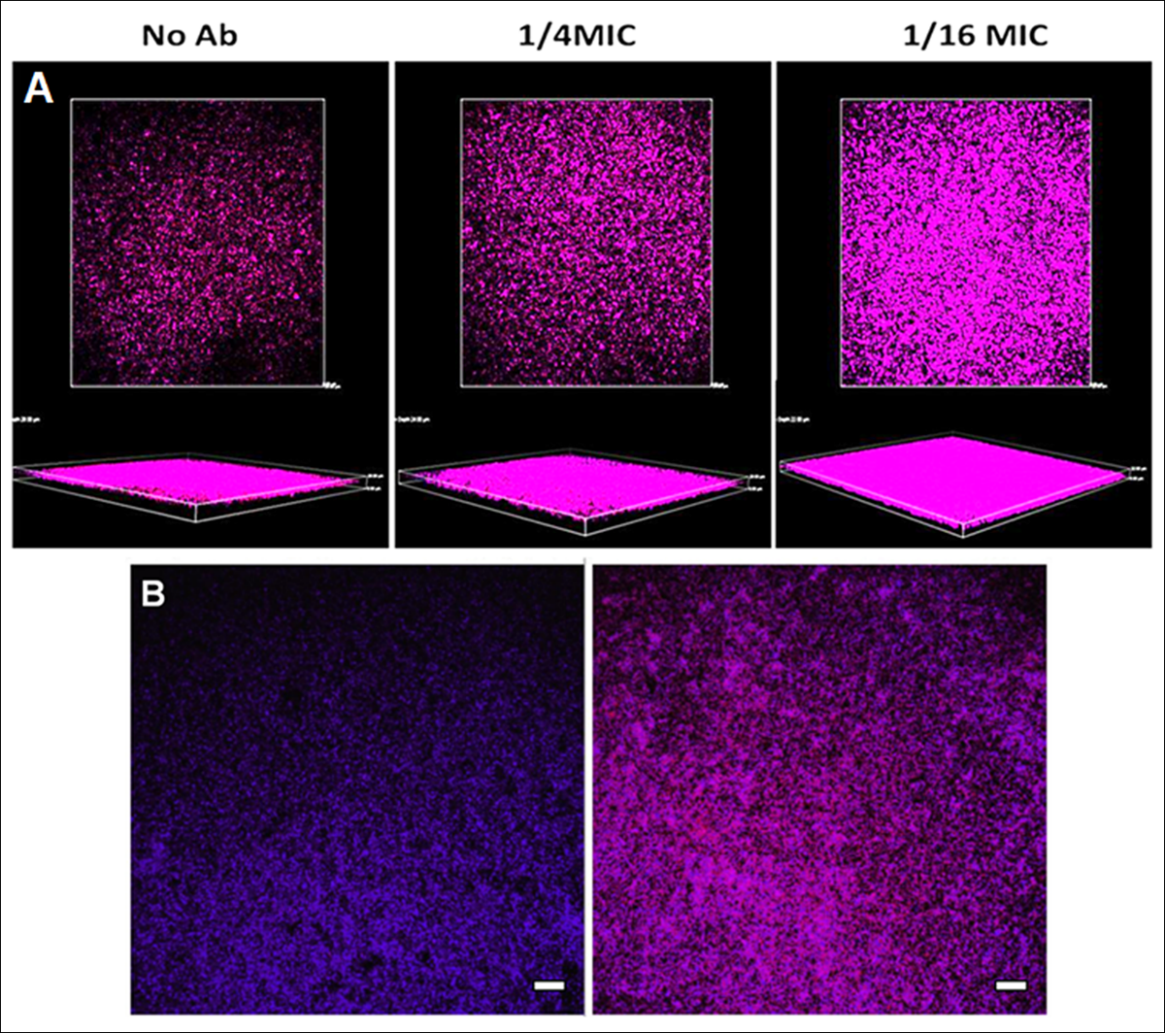
FilmMatrix and DNA staining. (A) coloured proteic matrix of strain 05/3291 exposed to different sub-MICs of ceftaroline. **No Ab**, without ceftaroline. (B) Biofilm composition of strains 06/1483 and 05/3291. CLSM of Film Tracer (purple) and NucBlue (blue) staining of strain**s** 06/1483 (left), and 05/3291 (right) without ceftaroline. Original magnification: ×200. Scale bars, 10μm.

Ceftaroline prevents MRSA biofilms at MIC (0.5 μg/ml). The assays indicate that although turbidity is not observed at the MIC, some bacteria can still survive at that concentration, as determined with the LIVE/DEAD staining (strain 06/1483). Our findings also showed that bacterial attachment and biofilm formation cannot be prevented by sub-MICs of ceftaroline (Fig 4). CLSM staining for matrix visualization in strains 06/1483 and 05/3291 are shown in Fig 5.

Strain 05/3291 shows an extracellular matrix. Importantly, at 1/4 MIC and 1/16 MIC of ceftaroline the matrix amount increased. Representative images of the effect of sub-MICs of ceftaroline on strain 05/3921 matrix are shown in Fig 5A. In accordance with our observations by SEM, CLSM staining shows that strain 06/1483 does not form an extracellular matrix (Fig 5B left).

The positive control (*P*. *aeruginosa*) which produces a dense and compact biofilm with abundant extracellular matrix was strongly stained with the FilmTracer (not shown).

### Gene expression

To study whether expression of the *icaA*, *agrA*, *sarA* or *sasF* virulence genes was modified by subinhibitory concentrations of ceftaroline in MRSA biofilms, we used quantitative real time PCR using primers described elsewhere. Our data show that these genes are present and likely functional in the strains used. The *agrA* and *icaA* genes were up-regulated in the ST125 strains at 1/4 MIC (fold regulation >2). Interestingly, these genes were consistently down-regulated in strain 05/3291 (ST5) with a fold regulation <-7 at 1/4 MIC of ceftaroline and with a fold regulation <-3 at 1/16 MIC; however, these results were not statistically significant (p>0.05) with respect to controls. No correlation between ceftaroline and enhanced biofilm formation was established at 1/16 MIC. Results are shown in Table 3.

**Table 3:** Gene Expression of *S*. *aureus* genes in biofilms.

Strain	Gene	Fold Regulation (comparing to controls)		P value(comparing to controls)	
		1/4 MIC	1/16 MIC	1/4 MIC	1/16 MIC
06/1483	*agrA*	**2.779**	-1.434	**0.001**	0.270
	*icaA*	**2.178**	**1.169**	**0.005**	**0.018**
	*sarA*	**2.094**	-1.242	**0.018**	0.280
	*sasF*	**2.123**	**1.027**	**<0.001**	**0.005**
05/2369	*agrA*	**1.125**	-2.025	**<0.001**	0.223
	*icaA*	**4.262**	1.682	**0.005**	0.259
	*sarA*	**-1.135**	-1.297	**0.021**	0.830
	*sasF*	**1.210**	-1.589	**<0.001**	0.786
05/3291	*agrA*	-1.225	-2.093	0.267	0.537
	*icaA*	-7.223	-3.725	0.443	0.965
	*sarA*	1.413	-2.025	0.200	0.638
	*sasF*	**-1.288**	-2.774	**0.004**	0.326

Significant results are highlighted in bold (p<0.05).

## Discussion

Ceftaroline is a broad-spectrum cephalosporin antimicrobial with extended gram-positive bacteria coverage. It exhibits greater binding affinity than ceftriaxone, oxacillin and penicillin G for PBPs in MRSA, with particular increased affinity for the PBP2a [31, 32]. Ceftaroline provides an important new treatment option that may help overcome some of the current challenges we face when managing patients with MRSA infections.

Numerous studies have shown that subinhibitory concentrations of some antibiotics can modulate bacterial biofilm formation *in vitro*. This process has clinical relevance because bacteria are exposed to sub-MIC concentrations of antibiotics at the beginning and end of a dosing regimen, between doses, or continuously during low-dose therapy [33]. Therefore, the effects of sub-MICs of antibiotics on microorganisms are of medical and biological interest. A few recent studies indicate that exposure of *S*. *aureus* to subinhibitory concentrations of some antibiotics may trigger an increase in some of its virulence factors and biofilm formation [17, 34, 35].

In this study, we investigated the effects of subinhibitory concentrations of ceftaroline on biofilm formation by MRSA strains. Unlike other studies using trypticase soy broth (TSB) culture medium for biofilm formation in *S*. *aureus*, we used for these assays Mueller-Hinton because it is the indicated medium to analyze antimicrobial susceptibility testing. To obtain consistent biofilms, 1% of glucose was added. The addition of this sugar did not alter the MICs for each strain. All the clinical strains tested were susceptible to ceftaroline, with MICs ranging 0.25–0.5 μg/ml. Electron micrographs taken after 48h exposure to ceftaroline showed breaks in the walls of several cells and ghosts of lysed cells. The morphological changes triggered by ceftaroline are in agreement with the PBP binding affinities of this antibiotic and these images represent a visual record of the bactericidal effect of ceftaroline. Interestingly enough is our observation of non-dividing cells in strain 05/3291 treated with ceftaroline, which are mainly broken (but not deformed) at the site where the peripheral wall ring leads to daughter cell separation in *S*. *aureus* (Fig 1 and S2 Fig). The impact of this finding should be in the research line of a recent paper, where Zhou and coworkers demonstrate that points of mechanical failure could initiate a propagating crack in this species [36]. We speculate on the possibility of harnessing this Aquilles heel to fight *S*. *aureus* strains similar to 05/3291.

Ceftaroline affected the biofilm formation of all strains tested at MIC, and in one strain (05/2369) at all concentrations tested. However, already at 1/2 MIC, biofilms formed by two strains were significantly higher (p<0.05) than biofilms formed in absence of antibiotic. Importantly, at lower sub-MICs of ceftaroline (from 1/4 to 1/16) the biofilm growth of two MRSA and the MSSA isolate was not affected.

Once biofilms are completely developed by some pathogens, conventional antibiotic treatments fail to eradicate the biofilm layers, and high biofilm-eradicating concentrations are required [37]. Due to this, strategies aimed at destroying biofilms acquire importance and therefore, the interaction between preformed biofilms of MRSA and ceftaroline was also studied in this work. Ceftaroline is able to disrupt preformed biofilms only in two strains (06/1483 and 05/2369) at MIC (0.5 μg/ml). Strain 05/2369 seems to be especially susceptible to ceftaroline also at 1/2 MIC (0.25 μg/ml). In other strains, a visible effect using 2×MIC (but not statistically significant) was quantified, including the MSSA strain. Interestingly, ceftaroline was not able to significantly reduce preformed biofilms in the MSSA strain (ATCC 29213) even at 4×MIC (0.5μg/ml). The biofilm formed by a matrix-producing strain 05/3291, was resistant even at 2×MIC. This strain belongs to a different MRSA clone, and, although all strains of the two different clones in our study showed similar MICs, strain 05/3291 produces a visible extracellular matrix enriched in proteins, well conserved and visible after processing for microscopy.

Very recently, Landini et al., [38] demonstrated that higher and repeated concentrations of ceftaroline exhibited a bactericidal activity against MRSA and MSSA biofilms. This biofilm destruction test is more similar to the *in vivo* situation, as bacteria have already colonized tissues or implanted biomaterials when an antibiotic is administrated to treat an infection. One of the advantages of CLSM is that it allows in-depth analysis of biological structures without killing or damaging the biological structure. The specific dye present in the Film Tracer^TM^ is able to mark most of the extracellular proteins in the biofilm such as fibrillar proteins, lipoproteins, phosphoproteins, and glycoproteins. Similar extracellular matrix was not observed in other MRSA strains in our study.

Several studies have demonstrated that *S*. *aureus* is able to produce proteinaceous biofilm matrices. Biofilms embedded in an extracellular matrix are largely protected from phagocytosis by neutrophils and macrophages. In a recent report, Gil et al., shows that an extract containing biofilm matrix exoproteins induces a protective immune response against an *S*. *aureus* biofilm-related infection and thus reduces colonization and persistence [37].

It has been suggested that the matrix of biofilms can be responsible for the increased resistance to antibiotics by acting as a diffusion barrier [39–42]. Importantly in our study, at 1/4 and 1/16 MIC of ceftaroline the proteinaceous matrix amount stained with fluorescence increased, and future studies will be required to determine if these preliminary findings in strain 05/2391 can be extended to larger numbers of matrix-producing MRSA clinical isolates. Moreover, this could mean that at low concentrations of ceftaroline an increased expression of genes related to the proteinaceous matrix is taking place.

The transcriptome analysis performed in MRSA strains after challenge by a sublethal concentration of tigecycline by Smith et al., shows the upregulation of genes encoding essential components of the protein synthesis pathway that may be part of the bacterial stress response in an attempt to withstand the antimicrobial challenge, as well as genes encoding several adhesins [11]. Therefore, we further compared the transcription expression of *agrA*, *icaA*, *sarA* and *sasF* genes in sub-MIC ceftaroline-treated cells of strains 06/1483, 05/2369 and 05/3291 with that of these genes in untreated cells, and found that there was a correlation between the regulation of *icaA* and *agrA*, and the increase of biofilms in presence of sub-MICs in strains 06/1483 and 05/2369 (both strains ST125) but there was not a correlation between the regulation of *icaA* and *agrA*, and the increase of biofilms in presence of sub-MICs in strain 05/3291 (ST5). On the other hand, no correlation between ceftaroline-enhanced biofilm formation and gene regulation was established at 1/16 MIC. Subrt et al. [34] showed that the β-lactam antibiotic cephalothin, when present at 1/4 MIC, induced *S*. *aureus* biofilm formation but did not affect *agr* expression. Consistent with these results, Kaplan et al. showed that an *agr* mutation did not affect the amount of biofilm induction caused by low-level methicillin in *S*. *aureus* [17]. On the other hand, Joo et al., showed that tetracycline, clindamycin, and other protein synthesis inhibitors at subinhibitory concentrations significantly increased the expression of *agr* in a community-associated MRSA strain [43]. Our results emphasize that antibiotics should be used in adequate dosages in order to avoid low subinhibitory concentrations, which can influence the gene expression pattern of bacteria in an unfavorable manner.

One way to overcome biofilm-associated resistance is through synergistic effects, created by the use of antimicrobial agents in combination, which can result in a rapid increase in antibiofilm activities. Results obtained by Barber et al., using ceftaroline combinations (daptomycin or vancomycin) are leading in the same direction [44]. Importantly, in our work, ceftaroline alone was very effective in reducing preformed biofilms in non-matrix producing strains. On the other hand, our results show that ceftaroline fail to reduce preformed biofilms in a matrix forming strain. Knowledge of specific antimicrobial activity against biofilm-forming staphylococci is an important determinant for choosing preventive or curative antimicrobial therapy, as well as MIC measurement against sessile cells (cells embedded in biofilm) and further studies are needed for investigation of the mechanism of biofilm induction in the presence of sub-MICs of ceftaroline.

## Supporting Information

S1 Fig 24-well microtiter plates after CV solubilisation. Examples of wells from 24-well microtiter plates after CV solubilisation. (A) glacial acetic acid (33%); (B) ethanol:acetone. Strains were grown on Mueller Hinton supplemented with 1% glucose for 48h at 37°C. C, controls (uninoculated wells).
(TIF)

S2 Fig SEM microphotographs of strain 05/3291 with 1/2 MIC ceftaroline. SEM microphotographs of strain 05/3291 in presence of ceftaroline (1/2 MIC). Cell surface is pseudocolored in purple and the cytoplasm in blue (right). Arrows indicate the point of fracture in non-dividing cells. Original Magnification: ×20.000.
(TIF)
