# Supplementary figures and images for: Effects of Subinhibitory Concentrations of Ceftaroline on Methicillin-Resistant Staphylococcus aureus (MRSA) Biofilms
Source: PLoS One. 2016 Jan 22;11(1):e0147569. doi: 10.1371/journal.pone.0147569 (PMC4723258; doi:10.1371/journal.pone.0147569)

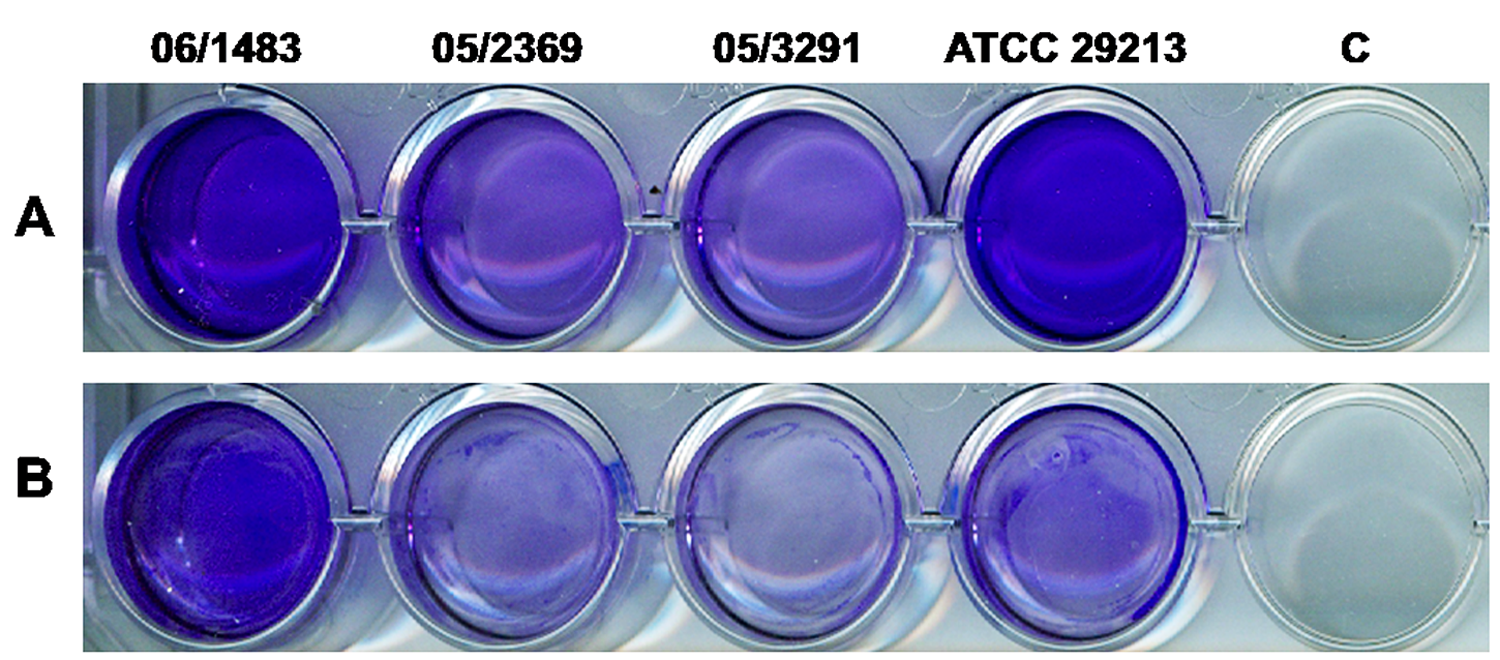

Supplement: S1 Fig — Examples of wells from 24-well microtiter plates after CV solubilisation. (A) glacial acetic acid (33%); (B) ethanol:acetone. Strains were grown on Mueller Hinton supplemented with 1% glucose for 48h at 37°C. C, controls (uninoculated wells). (TIF) [file pone.0147569.s001.tif]

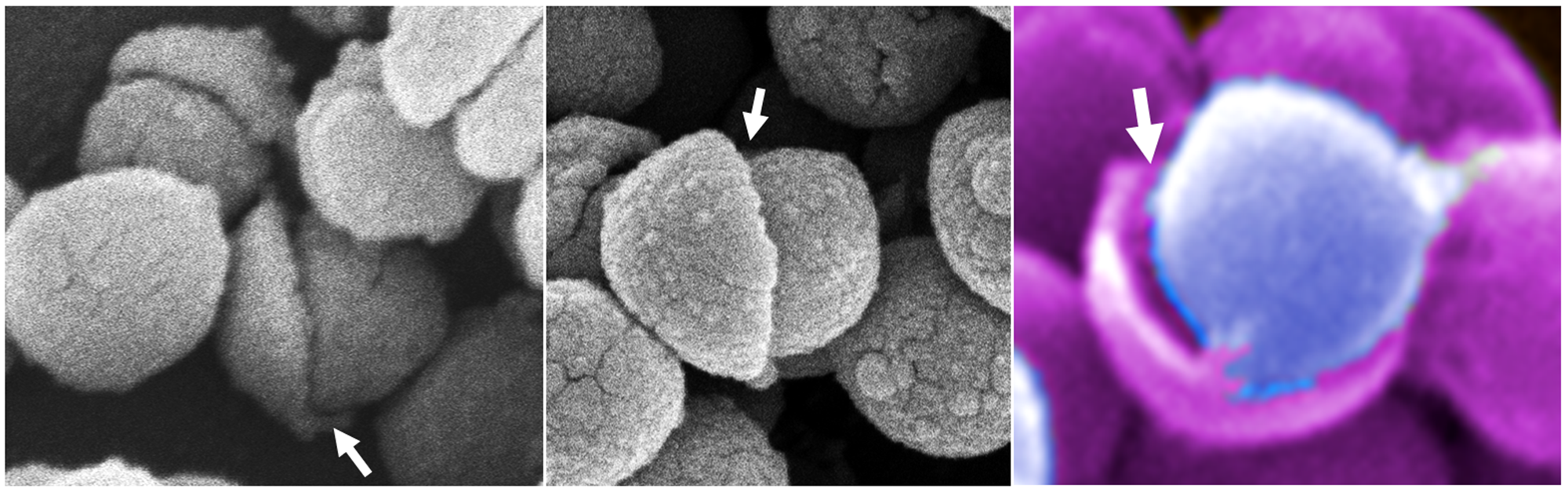

Supplement: S2 Fig — SEM microphotographs of strain 05/3291 in presence of ceftaroline (1/2 MIC). Cell surface is pseudocolored in purple and the cytoplasm in blue (right). Arrows indicate the point of fracture in non-dividing cells. Original Magnification: ×20.000. (TIF) [file pone.0147569.s002.tif]
